# Effects of yeast trehalose-6-phosphate synthase 1 on gene expression and carbohydrate contents of potato leaves under drought stress conditions

**DOI:** 10.1186/1471-2229-12-74

**Published:** 2012-05-30

**Authors:** Mihály Kondrák, Ferenc Marincs, Ferenc Antal, Zsófia Juhász, Zsófia Bánfalvi

**Affiliations:** 1 Agricultural Biotechnology Center, Szent-Györgyi Albert u. 4, Gödöllő, Hungary

## Abstract

**Background:**

The development of drought-tolerant, elite varieties of potato (*Solanum tuberosum* L.) is a challenging task, which might be achieved by introducing transgenic lines into breeding. We previously demonstrated that strains of the White Lady potato cultivar that express the yeast trehalose-6-phosphate synthase ( *TPS1*) gene exhibit improved drought tolerance.

**Results:**

We investigated the responses of the drought-sensitive potato cultivar White Lady and the drought-tolerant *TPS1* transgenic variant to prolonged drought stress at both the transcriptional and metabolic levels. Leaf mRNA expression profiles were compared using the POCI microarray, which contains 42,034 potato unigene probes. We identified 379 genes of known function that showed at least a 2-fold change in expression across genotypes, stress levels or the interaction between these factors. Wild-type leaves had twice as many genes with altered expression in response to stress than *TPS1* transgenic leaves, but 112 genes were differentially expressed in both strains. We identified 42 transcription factor genes with altered expression, of which four were uniquely up-regulated in *TPS1* transgenic leaves. The majority of the genes with altered expression that have been implicated in photosynthesis and carbohydrate metabolism were down-regulated in both the wild-type and *TPS1* transgenic plants. In agreement with this finding, the starch concentration of the stressed leaves was very low. At the metabolic level, the contents of fructose, galactose and glucose were increased and decreased in the wild-type and *TPS1* transgenic leaves, respectively, while the amounts of proline, inositol and raffinose were highly increased in both the wild-type and *TPS1* transgenic leaves under drought conditions.

**Conclusions:**

To our knowledge, this study is the most extensive transcriptional and metabolic analysis of a transgenic, drought-tolerant potato line. We identified four genes that were previously reported as drought-responsive in non-transgenic Andean potato cultivars. The substantial increases in proline, inositol and raffinose contents detected in both the wild-type and *TPS1* transgenic leaves appears to be a general response of potatoes to drought stress. The four transcription factors uniquely up-regulated in *TPS1* transgenic leaves are good candidates for future functional analyses aimed at understanding the regulation of the 57 genes with differential expression in *TPS1* transgenic leaves.

## Background

One major factor limiting food production worldwide is soil water deficits. Currently, 70% of the world’s total water consumption is used for agriculture. However, the 18% of agricultural land that is irrigated produces approximately 40% of the global food supply. The rest of the food supply relies on natural water resources such as floods and rain. The amount of food required by the developing world is expected to double by 2025. It is likely that this enormous increase in production will take place on the same or even a decreased land area, with less water available due to the effects of global climatic change [1]. These negative effects, however, will influence not only arid and semi-arid regions but also continental climate areas. Thus, developing appropriate varieties, agricultural practices and management strategies to produce crops under drought stress will be a challenge for the 21st century.

Potato (*Solanum tuberosum* L.) is the third most important food crop in the world, with annual production exceeding 300 million tons (http://faostat.fao.org). Compared to other crops, potato is considered drought sensitive, and even short periods of stress can cause significant reductions in tuber yield [2]. Recent advances in understanding the genetic control of drought tolerance offer new opportunities to develop crops that are less damaged by low soil moisture. These advances could prevent or reduce crop losses and decrease the amount of water needed for irrigation, which is an important goal for areas with increasingly limited water supplies [1].

Potato drought tolerance has been addressed at the morphological, physiological and molecular levels. Canopy architecture, root size, photosynthesis, and sugar accumulation-related traits are associated with drought tolerance in this species [3-8]. Gene expression and metabolite profiling revealed drought tolerance candidate genes involved in cell signalling, elimination of reactive oxygen species, biosynthesis of long-chain fatty acids and waxes, enhanced production of cell-protective factors such as LEA and heat shock proteins, and osmolyte accumulation [9-13].

One way to modify plant water usage is to genetically engineer drought-tolerant strains. Many organisms have evolved traits that enable them to survive in extreme environments, and the gene(s) underlying these phenotypes could potentially be introduced into crop plants. Some of these genes encode stress proteins, which are directly implicated in stress tolerance, while others encode proteins involved in the synthesis of osmolytes [14]. In the potato, osmotic adjustment is associated with increased concentrations of sucrose, raffinose, galactinol, pinitol, proline and polyamines [11]. Trehalose (α,α-1,1-di-glucose) is one such osmolyte that can adjust osmosis and protect macromolecules [15]. A number of genes involved in trehalose metabolism, including the yeast trehalose-phosphate synthase 1 (*TPS1*) gene, have been used to improve the drought tolerance of several different plant species [16].

To obtain drought-tolerant potato plants, we previously transformed *S. tuberosum* cv. White Lady with the yeast *TPS1* gene driven by the drought-inducible potato promoter *StDS2*. *TPS1* transgenic lines were drought-tolerant: they displayed higher stomatal conductance and net photosynthesis rates than wild-type plants under drought stress, and their detached leaves wilted more slowly than leaves of control plants [17]. Stress-inducible promoters usually maintain a low level of expression of the regulated gene, even under non-inducing growth conditions. However, that low expression level may have negative pleiotropic effects on the plant under conditions where the product of the expressed gene is not necessary [14]. We have observed such negative pleiotropic effects in the case of the *TPS1* transgenic lines, which displayed stunted growth, a significant, on average 30% reduction in shoot mass and leaf area, a lower CO_2_ fixation rate and reduced stomata number compared to wild-type plants under well-watered conditions [17]. To understand the molecular basis of this phenomenon, we compared the transcriptomes of wild-type and *TPS1* transgenic plants. We shown that 74 and 25 genes were up- and down-regulated, respectively, in the mature source leaves of *TPS1* transgenic plants compared to wild-type controls. We also demonstrated that the starch content was lower, while the malate, inositol and maltose levels were higher in *TPS1* transgenic than wild-type leaves [18].

Despite the negative effects caused by the expression of the *TPS1* gene, the transgenic lines did display drought stress tolerance [17]. In this study, we describe the results of genome-wide transcriptional profiling and metabolic analyses of *TPS1* transgenic and wild-type potato leaves under drought stress and define differences and similarities in gene expression and metabolite content of natural, introgressed, and transgenic drought-tolerant lines.

## Results

### The effect of drought on the potato leaf transcriptome

To compare the leaf transcriptomes of drought-tolerant *TPS1* transgenic and drought-sensitive wild-type (WT) potato plants, samples of each line were grown under irrigation or drought stress in a greenhouse. After two weeks of water restriction, we collected source leaves and determined the relative water content (RWC) of one composite leaf per plant to ensure that the water status of the plants was in the stage that reflects the phenotypic and physiological differences between the drought-tolerant *TPS1* transgenic and the drought-sensitive WT control plants [17]. The RWC values of the lines were the same under well-watered conditions (85 ± 1%), but under drought stress, the RWCs of the *TPS1* transgenic T1 and T2 lines were markedly higher (70 ± 8% and 81 ± 1%, respectively) than the RWC of the control WT leaves (65 ± 7%). These values correspond to previously determined values [17]. RNA was isolated from the rest of the leaves of each line. For microarray analysis, however, we used only the T2 line because this line was previously used for a microarray analysis of plants grown under well-watered conditions [18].

**Figure 1:**
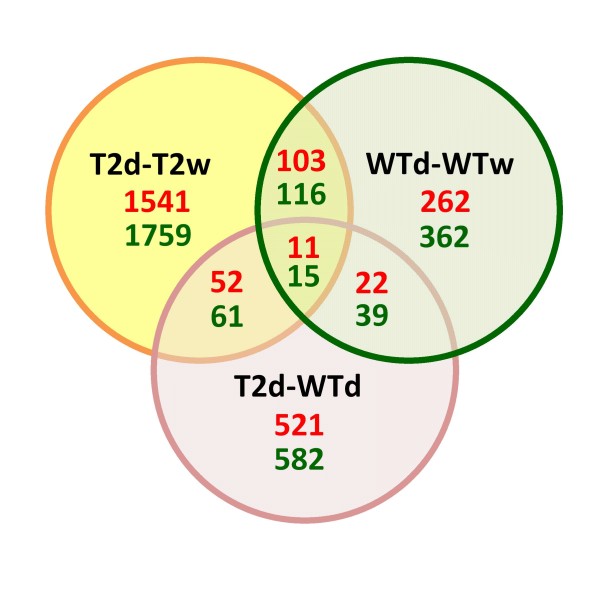
**Venn diagram showing the number of differentially expressed genes in response to drought stress.** T2d, *TPS1* transgenic line T2 under drought stress; T2w, *TPS1* transgenic line T2 under irrigation; WTd, wild-type plant under drought stress; WTw, wild-type plant under irrigation. The numbers of up-regulated genes are shown in red, while the numbers of down-regulated genes are shown in green.

Total RNA isolated from the leaves was transcribed into fluorescently labelled cDNA, which was then hybridised to 60-mer oligonucleotide potato microarrays [19] as previously described [18]. The following three comparisons were performed: WT plants under drought versus WT plants under irrigation (WTd-WTw), T2 plants under drought versus T2 plants under irrigation (T2d-T2w), and T2 plants under drought versus WT plants under drought (T2d-WTd). All comparisons were performed with three biological replicates and three technical replicates. Normalised data from the three comparisons were subjected to statistical analysis using the “Rank products” method [20] with which we identified 5,446 genes with statistically significant (*P ≤* 0.05) changes in expression level. The very large majority (95.72%) of these genes had an expression ratio larger than two-fold and the number of genes with statistically significant changes was about four-times more in the T2d-T2w than in WTd-WTw experiment (Figure 1).

**Figure 2:**
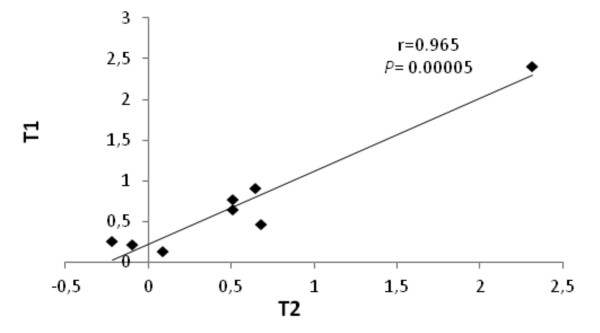
**Linear regression analysis between qRT-PCR data obtained from the independent*TPS1*transgenic lines T1 and T2.** Log2 ratios are presented on both axes. The following genes were analysed: ribulose bisphosphate carboxylase small chain 2B, fructose bisphosphate aldolase, ETHYLENE-INSENSITIVE3-like 1 transcription factor, a bZIP transcription factor family protein, EMBRYO DEFECTIVE 2220 transcription factor, a plant homeodomain finger family protein, a universal stress protein, and *StDS2*, a drought-inducible potato gene.

To validate the microarray data, eight genes ( Additional file 1) were selected for quantitative real-time polymerase chain reaction (qRT-PCR) analysis. This analysis was performed for all three comparisons with three replicates each. The expression ratios of the analysed genes showed positive correlations between the microarray and qRT-PCR analyses in all three experiments. Correlation coefficient (r) values were 0.662, 0.728, and 0.678 at the *P ≤* 0.04 level for the WTd-WTw, T2d-T2w, and T2d-WTd experiments, respectively, indicating the reliability of the microarray data. As only one of the *TPS1* transgenic lines, T2, was used for the microarray analysis, we compared the expression of the eight selected genes between the T1 and T2 lines by qRT-PCR and found that they yielded high positive correlations in comparisons of transgenic drought-stressed versus transgenic irrigated plants (Figure 2).

To reduce the large number of genes (5,446, see above) with statistically significant differences in expression level and give a biological sense to them, normalised microarray data were also analysed by a two-way ANOVA to determine the effects of genotype (T2 versus WT) and stress (drought versus irrigated). In this analysis, only the WTd-WTw and T2d-T2w comparisons were included because these had a two by two factor setup, a prerequisite for two-way ANOVA. This analysis revealed 1,496 genes for which expression depended on genotype, stress or their interaction at the *P* ≤ 0.01 level. It is worth noting that all 1,496 genes could be found amongst the 5,446 genes displaying statistically significant changes in expression indicating the concordance and reliability of the employed statistical analyses. Moreover, all genes returned by ANOVA had an expression ratio equal/larger than two, indicating the power of the analysis. The 1,496 genes were subjected to functional annotation using the MapMan software. Of the 1,496 genes, 487 were annotated into the “not assigned” bin. We note that 35 of the remaining 1,009 genes were also assigned into that bin but do encode known proteins. Selection the common genes between the ANOVA- and Mapman-returned gene sets yielded a total of 379 genes ( Additional file 2) of known function that were differentially expressed (minimum 2-fold) in a manner dependent on genotype, stress or the interaction between the two factors, and we discuss these selected genes below. The drought-inducible gene *StDS2*[21], whose promoter was used to express the *TPS1* transgene [17], was highly induced by drought, as indicated by the 3.1 and 5.1 log2 expression ratio values obtained in the T2d-T2w and WTd-WTw comparisons, respectively. *StDS2* expression was also found to be stress-dependent ( *P* = 0.007547) in the two-way ANOVA. Nevertheless, *StDS2* did not appear among the 379 genes, as it was not assigned into any functional category by the MapMan software.

### Functional categories of the differentially expressed genes

The MapMan software, which we used for functional annotation, categorises potato genes into 36 bins. Overall, the 379 genes were classified into 30 functional categories out of the possible 36 bins ( Additional file 3). Genes in the general regulation category (transcriptional, translational, and post-translational regulation, signalling and transport) were highly represented; their proportion varied between 44 (T2d-T2w, down-regulated genes) and 52% (WTd-WTw, up-regulated genes). According to the two-way ANOVA results, expression of the genes in the regulation category was almost exclusively stress-dependent. In the other broad functional categories, metabolism was represented at almost uniform proportions amongst the down- and up-regulated genes in both the T2d-T2w and WTd-WTw comparisons. However, the number and proportion of lipid and secondary metabolism genes were slightly higher amongst the up-regulated genes in both comparisons relative to other types of metabolism. There was no significant difference between the T2 and WT lines in the relative proportions of functional categories of genes down-regulated by drought stress. In contrast, there was a marked difference in the proportions of up-regulated genes, i.e., almost twice as many genes implicated in protein synthesis, post-translational modification or degradation were found in drought-stressed WT relative to drought-stressed T2 plants (Additional file 3).

### Comparing the drought stress response in the leaves of *TPS1* transgenic and wild-type plants

**Figure 3:**
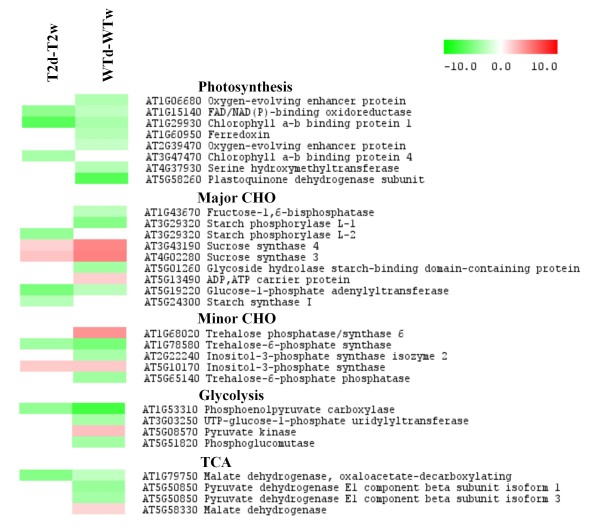
**Heatmap of differentially expressed genes associated with photosynthesis and carbohydrate metabolism.** The expression ratios of the genes in the T2d versus T2w and WTd versus WTw comparisons are shown as coloured rectangles and were visualised in the Multiple Experiment Viewer (MeV) software. The colour scale indicates the expression ratios as log2 values, with red and green colours for up- and down-regulated genes, respectively. T2d, *TPS1* transgenic line T2 under drought stress; T2w, *TPS1* transgenic line T2 under irrigation; WTd, wild-type plant under drought stress; WTw, wild-type plant under irrigation.

Photosynthesis and cell growth are among the primary processes affected by water deficits [22]. In line with this general observation, genes associated with photosynthesis were down-regulated in the drought-stressed WT and T2 plants. The number of genes affected, however, was higher in WT than in T2 samples, indicating a milder effect of drought on T2 plants (Figure 3).

Genes encoding starch phosphorylase, which is implicated in phosphorolytic degradation of starch, were down-regulated in drought-stressed WT and T2 plants. Glycoside hydrolase, which cleaves glycoside bonds to release simple sugars, was also down-regulated in WT leaves. Interestingly, drought stress induced the expression of two sucrose synthase isoforms, *SUS3* and *SUS4*, in both WT and T2 leaves (Figure 3). In potato plants grown under optimal conditions, *SUS3* genes are most highly expressed in stems and roots and appear to provide the vascular function of sucrose synthase, while *SUS4* genes are primarily expressed in the storage and vascular tissue of tubers and appear to facilitate the sink function [23].

In Arabidopsis, trehalose-6-phosphate synthases (TPSs) and trehalose-6-phosphate phosphatases (TPPs), encoded by different classes of *TPS* and *TPP* genes, are differentially expressed in response to a variety of abiotic stresses [24]. Drought may have a similar effect in potato because we found that one *TPS-TPP* gene was up-regulated and one *TPP* gene was down-regulated in WT leaves, while another *TPS* gene was down-regulated in both T2 and WT leaves (Figure 3).

The TCA cycle, which is part of a metabolic pathway that generates energy by converting carbohydrates, fats, and proteins into carbon dioxide and water, may be repressed by drought stress. This conclusion is supported by the observation that drought stress had a negative effect on the expression of genes encoding malate-dehydrogenase and pyruvate dehydrogenase, which are involved in the TCA cycle and link glycolysis to the TCA cycle, respectively. In addition to the TCA cycle, glycolysis may also be repressed in WT leaves because three genes encoding enzymes involved in glycolysis were down-regulated, and only one gene was slightly up-regulated. Compared to WT, drought stress had a milder effect on both the TCA cycle and glycolysis in T2 leaves (Figure 3).

**Figure 4:**
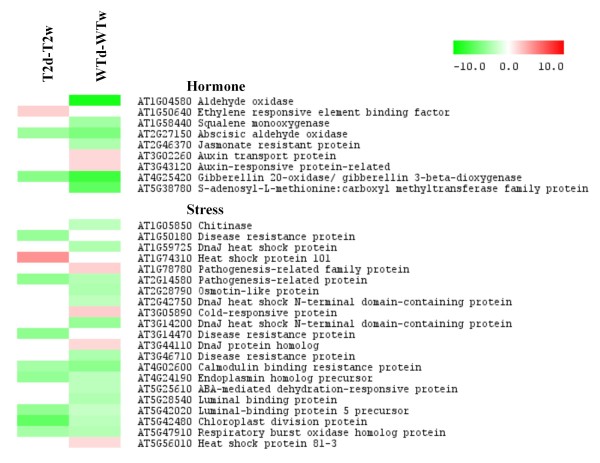
**Heatmap of differentially expressed genes associated with hormone metabolism and the stress response.** Labels are as in Figure 3.

Drought stress affected the expression of three and eight genes involved in hormone metabolism in T2 and WT plants, respectively; most of these genes were down-regulated. Unexpectedly, most of the genes in the stress-related functional group were down-regulated in both T2 and WT leaves. Only one heat shock protein gene showed stress-induced expression in T2 plants, whereas genes encoding a heat-shock protein, a pathogenesis-related protein, and a DnaJ homolog protein were up-regulated by drought in WT plants (Figure 4).

A relatively large number (42) of transcription factor (TF) genes showed altered expression in either T2 or in WT plants under drought stress ( Additional file 4). These TFs belong to a number of different categories, and most showed the same direction of regulation in T2 and WT leaves. In addition to the up-regulation of an auxin transport protein and an auxin-responsive protein, an auxin-induced TF gene was up-regulated in WT leaves, suggesting that the auxin level in leaf cells might have been increased by water depletion. The TFs up-regulated uniquely in T2 plants included a jumonji family protein (transcriptional repression and/or chromatin regulation [25]), a RNA polymerase σ70-type initiation factor (plastid genome transcription [26]), a NAC transcription factor (involved in jasmonate responses [27]), and a homeodomain family protein belonging to the PHD finger subgroup (chromatin modification and mediation of molecular interactions in gene transcription [28]).

Twenty-four genes, mainly receptor-like kinases and calcium-binding proteins with signalling functions, were down-regulated by drought in both WT and T2 leaves. In contrast, only four genes in the signalling category showed drought-inducible expression: two genes in T2, one in WT, and a glutamate receptor gene in both lines ( Additional file 5).

### Metabolite changes in drought-stressed leaves

**Figure 5:**
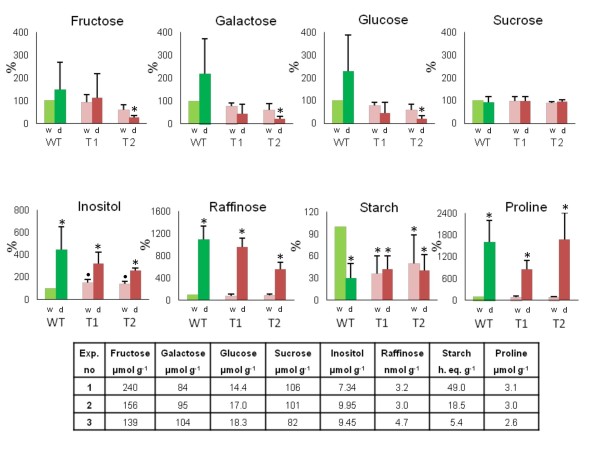
**Relative amounts of sugars, starch and proline in wild-type (WT) and*TPS1*transgenic (T1, T2) leaves compared to the well-watered WT control.** Bars and error bars represent the mean ± SE derived from three independent experiments. Asterisks and dots denote significant differences at the *P* = 0.01 and *P* = 0.05 ( *t* test) levels, respectively, as compared with the well-watered WT control. The absolute concentration of the different compounds in well-watered WT leaves in three independent experiments is shown in a table. Concentrations are calculated relative to dry weights (DW). The concentration of starch is given in hexose equivalents (h. eq.) g^-1^ DW. The high variation in leaf metabolite and starch content might be explained by the small differences in day length, light intensity and temperature in the greenhouse during the three consecutive plant tests, as environmental conditions can strongly influence metabolite content and starch accumulation in leaves [30]. Therefore, concentrations of compounds in the well-watered WT leaves were regarded as 100% for comparison with other samples originated from the same plant test. Mean ± SE of the percentage values obtained from the three consecutive plant tests were calculated and are presented by bars and error bars.

We previously showed that under well-watered conditions, the amounts of fructose, galactose, glucose, sorbitol, and sucrose were largely similar between the WT and *TPS1* transgenic lines, while the levels of inositol, maltose, and malate were higher in the *TPS1* line [18]. We extracted carbohydrates from the same pool of stressed leaves used for microarray analysis and found that the amounts of maltose and malate were the same as in irrigated plants (data not shown). We note here that the unchanged amount of malate was surprising because it has been shown that addition of malate reduces both activity and transcript level of nitrate reductase [29]. Since we have detected a strong down-regulation of nitrate reductase in both WT and *TPS1*-transgenic plants under drought stress ( Additional file 3), we expected an increase in malate concentration under stress. However, the levels of fructose, galactose, and glucose were higher in the stressed relative to the well-watered WT control leaves. In contrast, drought stress either did not change or even reduced the amounts of these three compounds in the *TPS1* transgenic lines. A 4.4-fold increase in the inositol level was detected in the drought-stressed WT leaves relative to the unstressed WT controls, while this increase was only 3.2-fold in T1 and 2.6-fold in T2 leaves. Considering that the inositol content was 1.6- and 1.4-fold higher in the T1 and T2 transgenic lines relative to the WT leaves under well-watered conditions [18], the actual increase in inositol level triggered by stress was only about 2-fold. Very strong 11-, 9.5-, and 5.5-fold increases in raffinose content were observed in response to drought treatment in WT, T1 and T2 leaves, respectively. Nevertheless, the concentration of raffinose was still quite low compared to other sugars, which were present in the μmol g^−1^ dry weight range, while the maximum concentration of raffinose was 50 nmol g^−1^ dry weight (Figure 5).

Drought stress reduced the starch content of the WT leaves by about 65%. In contrast, no significant change in the otherwise lower amount of starch in *TPS1* transgenic plants under the no-stress condition [18] was detected in the leaves in response to drought stress (Figure 5).

Proline accumulates in many plant species in response to environmental stress [31]. This prompted us to measure the concentration of proline in WT and *TPS1* transgenic plants. Proline increased by 10- to 18-fold in each line (Figure 5). In this respect, there is no difference in the responses of WT and *TPS1* transgenic lines to drought stress.

## Discussion

The microarray study described herein primarily focused on the leaf transcriptomes of the potato cultivar White Lady (WT) and the *TPS1* transgenic derivative (T2) exposed to drought stress in the form of 30% soil moisture content. By analysing the microarray data, more than 5,000 genes, which had statistically significant changes in their expression, were identified in the WT plants under drought versus WT plants under irrigation (WTd-WTw), T2 plants under drought versus T2 plants under irrigation (T2d-T2w), and T2 plants under drought versus WT plants under drought (T2d-WTd) comparisons. Although the stress treatment resulted in higher water loss in the drought-sensitive WT plants relative to the drought-tolerant *TPS1* transgenic plants, many more genes showed altered expression in response to stress in T2 than in WT leaves (3,658 versus 930 genes, respectively, Figure 1).

One major challenge in microarray analysis is to give biological sense to statistically significant data. In our work, we used three approaches to achieve this. First, we analysed our data by two-way ANOVA to identify genes whose expression depend on two factors, plant genotype (wild-type and transgenic) or treatment (drought stress and irrigation), or on the interaction of these two factors. By this analysis, we reduced the number of genes from 5,446 to 1,496. All genes returned by ANOVA had an expression ratio larger than two. Second, this reduced set of genes was annotated into functional categories using the MapMan software. The annotation returned 1,009 genes with known association with biochemical pathways or regulatory functions. Third, we identified the common genes between the ANOVA- and MapMan-returned sets. This resulted in 379 genes, which fulfilled all of the following criteria: (i) gene expression depends on either genotype or treatment or on the interaction of the two, (ii) they have a known function and (iii) the expression ratio is larger than two and is statistically significant at the *P* ≤ 0.05 level. We consider these 379 genes to have biological importance in drought physiology of potato.

Out of the 379 genes with altered expression, 112 were regulated in the same direction in response to drought in WT and T2 plants. Because the relative water content (RWC) of the T2 plants was only reduced from 85% to 81%, it appears that the expression of these genes is particularly sensitive to water loss. An alternative explanation is that the plant senses the water content of the soil and regulates the transcription of these genes accordingly. The 112 commonly regulated genes included nine down-regulated and three up-regulated genes involved in photosynthesis and carbohydrate metabolism, including chlorophyll a-b binding proteins, fructose-1,6-bisphosphatase, trehalose-6-phosphate synthase (all down-regulated), and sucrose synthase (up-regulated). Recently, Evers et al. [32] compared two potato clones of the Andean cultivar group with different drought tolerance phenotypes. Although the RWC of leaves exposed to prolonged drought stress was reduced by only 2–3%, repression of chlorophyll a–b binding proteins, fructose-1,6-bisphosphatase, and trehalose-6-phophate synthase and induction of sucrose synthase genes occurred in both Andean cultivars as well.

Induction of sucrose synthase 3 (*SUS3*) occurred not only under stress conditions in the WT plants (this study) but also in well-watered *TPS1* transgenic plants [18]. Furthermore, an ATP-dependent caseinolytic protease (an essential housekeeping enzyme in plant chloroplasts [33]), actin 7 (a structural constituent of the cytoskeleton [34]), and a V-type proton ATPase gene (an enzyme that transforms the energy from ATP hydrolysis to electrochemical potential differences in proton concentrations across diverse biological membranes [35]) were up-regulated in irrigated *TPS1* transgenic plants [18] and induced by stress in WT plants. There might therefore be a common signal generated by the expression of *TPS1* and drought stress that leads to the up-regulation of these four genes.

Evers et al. [32] reported that while biochemical changes did not clearly reflect gene expression changes in Andean cultivars, galactose, inositol and galactinol contents were higher in the drought-stressed tolerant cultivar relative to the more sensitive strain. Although we were also unable to directly correlate transcriptional changes with biochemical differences, we found an increase in galactose content in the sensitive WT plants and elevated inositol contents in both WT and *TPS1* transgenic plants. We also observed a 65% reduction in the starch content of WT leaves but no dramatic changes in sucrose in either line (Figure 5). The starch content of the *TPS1* transgenic leaves was not reduced in the drought-stressed plants but remained at the same low level as observed under well-watered conditions. We therefore speculate that a constant sucrose level may be very important for potato plants. Since stress reduces the rate of photosynthesis maintenance of a constant sucrose level under drought stress conditions may require the plants to reduce starch synthesis and channel the carbohydrates to sucrose synthesis.

Inositol is a versatile compound that generates diversified derivatives upon phosphorylation. These compounds have dual functions as signalling molecules as well as key metabolites under stress [36]. We previously found a 1.4- to 1.6-fold increase in the inositol level of leaves of *TPS1* transgenic plants grown under well-watered conditions. This elevation was further increased by drought to 2.6- to 3.2-fold higher than the well-watered WT control. In WT plants, a 4.4-fold increase in inositol content was detected in response to drought. Because the high level of inositol correlates with the low level of starch, we assume that inositol serves as a signal for the reduction of starch synthesis. Besides phosphatidylinositol, inositol-derived galactinol and associated raffinose family oligosaccharides are emerging as antioxidants and putative signalling compounds [36]. In a comparison of the carbohydrate metabolism of a drought-tolerant advanced potato clone and a sensitive commercial variety, the tolerant clone presented an increase in galactinol and raffinose contents, especially in the leaves [37]. We also found a very robust increase (5.5- to 11-fold) in raffinose content that was more pronounced in WT than *TPS1* transgenic plants. Unlike inositol, the raffinose level was not elevated under well-watered conditions in the *TPS1* transgenic lines compared to WT plants (data not shown). The regulatory mechanisms that underlie these increases in inositol and raffinose contents are likely quite different. While inositol synthesis is influenced by the transcriptional and/or biochemical changes triggered not only by drought but also by the expression of yeast *TPS1* in potato, raffinose synthesis is induced by water loss and is negatively correlated with leaf RWC.

Drought stress induced the accumulation of proline in both WT and *TPS1* transgenic leaves. Plant proline concentrations are regulated by an interplay of biosynthesis, degradation and intra- as well as intercellular transport processes. Proline is synthesised from glutamate or ornithine, and the first pathway initiated by Δ^1^-pyrroline-carboxylate reductase (P5CR) is considered to be dominant under stress conditions [38]. In Andean potato cultivars, the increase in proline was linked to the up-regulation of *P5CS* and the down-regulation of proline dehydrogenase ( *PDH*), which is involved in proline catabolism [32]. In our experiments, *P5CS* and *PDH* were not among the 379 selected genes, suggesting that other processes than transcriptional regulation might also influence the accumulation of proline in leaf cells.

We identified 57 genes with differential expression in T2 but not WT leaves. This difference in expression might be explained by the different RWCs of drought-stressed WT (65 ± 7%) and T2 (81 ± 1%) leaves but could also be attributed to transcriptional and metabolic changes induced by the transgene under well-watered [18] as well as drought stress conditions. We found four TFs uniquely up-regulated in T2 leaves: two different proteins involved in chromatin modification, one involved in plastid genome transcription, and one involved in jasmonate responses. Because TFs generally influence the transcription of a set of genes, it is possible that the four TFs alter the expression of several target genes and trigger a cascade of downstream signalling events.

Several different sets of *cis*- and *trans*-acting factors are known to be involved in stress-responsive transcription. Some are controlled by the phytohormone abscisic acid (ABA), but others are not, indicating the involvement of both ABA-dependent and ABA-independent regulatory systems for stress-responsive gene expression [39]. Expression of *StDS2* is highly drought-specific and independent of ABA [21]. In this study, induction of *StDS2* expression was detected in both WT and *TPS1* transgenic leaves. Surprisingly, however, ABA-responsive marker genes such as *RD22ERD15* and *ERD3* did not appear in our selected list of genes. Instead, we found that abscisic aldehyde oxidase, which catalyses the last step of ABA biosynthesis, and an ABA-mediated dehydration-responsive protein transcript were down-regulated in both WT and T2 and only WT plants, respectively (Figure 4). Together, these correlative changes suggest that the ABA level after prolonged drought stress is not as high as observed in short-term responses to osmotic stress, although this has yet to be directly verified.

## Conclusions

This work aimed to compare the responses of wild-type and drought-tolerant transgenic potato lines to drought conditions at the transcriptional and metabolic levels. As the result of microarray data analysis, we identified 379 genes in the leaf transcriptome, which belong to 30 functional categories, their expression depend on genotype, drought stress or the interaction between the two, and their expression ration is at least two-fold. Transcription factor genes represented about 11% of these genes. The number of down-regulated genes was about 2-fold higher than the number of up-regulated genes, indicating that cells switch to an economic state in response to drought conditions. We identified 112 genes that were up- or down-regulated in both WT and T2 plants. Because the water content of the drought-stressed T2 leaves was only slightly lower under stress than under well-watered conditions, we presume that the expression of these genes is either very sensitive to water loss or, more likely, depends on the availability of soil water. This conclusion is supported by the fact that several of these 112 genes responded to drought stress in the same way in our potato lines as in clones of Andean potato cultivars [32]. Fifty-seven genes, including four up-regulated TFs, showed altered expression only in *TPS1* transgenic plants. These TFs are good candidates for functional analyses aimed at understanding the regulation of the 57 genes that only showed differential expression in T2 leaves. We also identified three auxin-responsive genes that were up-regulated only in drought-stressed WT leaves, suggesting that the auxin level is increased by stress in WT but not T2 leaves. No ABA-responsive marker genes appeared in our gene list. We therefore conclude that the ABA level may not be as high after prolonged drought stress as observed upon short-term osmotic stress. Finally, we found four genes that were up-regulated in irrigated *TPS1* transgenic plants [18] and were induced by stress in WT plants, indicating that *TPS1* expression can generate a signal common with drought stress.

Although the biochemical changes that we detected did not clearly reflect the changes in gene expression, we found three compounds, inositol, raffinose, and proline, that were highly increased by drought in both WT and *TPS1* transgenic leaves, as previously observed in other potato cultivars [32,37]. The starch content of WT leaves was strongly reduced by drought, while that of the *TPS1* transgenic leaves remained at the same low level as observed under well-watered conditions. We found that the high level of inositol correlated with the low level of starch, suggesting that inositol, which is thought to have a signalling function in the stress response [36], is involved in the reduction of starch synthesis. Nevertheless, this suggestion needs further experimental verification. While the inositol content of *TPS1* transgenic leaves was elevated, even in unstressed plants [18], the raffinose and proline contents were only increased under drought stress. We conclude that inositol synthesis is influenced by transcriptional and/or biochemical changes triggered not only by drought but also by the expression of yeast *TPS1*. In contrast, raffinose and proline synthesis were drought-specific and induced either by leaf water loss or by sensing the low soil moisture. Nevertheless, the elevated levels of raffinose and proline did not prevent the plants from wilting because drought stress induced high levels of these compounds in both the drought-tolerant *TPS1* transgenic plants and the drought-sensitive WT plants.

## Methods

### Plant material and growth conditions

*In vitro* plantlets of *Solanum tuberosum* cv. White Lady and their *TPS1* transgenic progeny, T1 and T2 [17], were propagated from nodal cuttings. Plantlets were maintained for six weeks in 30-ml test tubes on RM medium [40] at 24°C under 90 μE m^−2^ s^−1^ and a 16 h light / 8 h dark photoperiod. The plantlets from *in vitro* culture were transferred to 3,000 ml pots containing A260 sterile soil (Stender, Germany) and grown in a greenhouse at 20–28°C under long day conditions and 70% soil water content. Four weeks after planting into soil, the plants were divided into two groups. Three plants per line were continuously irrigated to maintain 70% soil moisture content, while three plants per line were exposed to a uniformly ramped drought stress by withholding irrigation. To do this, soil drying was monitored by weighting the pots daily and only that much water was added to them that they all got the same weight as the heaviest pot. This process was continued until the water content of the soil of the stressed plants decreased to 30%, and then it was maintained at that level. Two weeks after starting the drought stress, all of the mature source leaves from three plants of each line-treatment combination were collected four hours after sunrise. One composite leaf per plant was used to determine the relative water content (RWC) using the following equation: RWC = (FW – DW) x 100 / (SW – DW), where FW is the fresh weight, SW is the water-saturated (turgid) weight and DW is the dry weight after drying for 24 h at 80°C. The rest of the collected leaves were ground in liquid nitrogen and kept at −70°C for RNA and metabolite isolation. The entire process was independently repeated three times to obtain three biological replicates for each line.

### RNA isolation, cDNA synthesis, microarray processing and qRT-PCR

For the transcriptome analysis, leaf samples were collected from plant lines grown in three biological replicates under well-watered and drought stress conditions as described above. Sample pooling, RNA isolation, cDNA synthesis and microarray processing were performed as published previously [18].

Validation of the microarray results was carried out by qRT-PCR. Two micrograms of DNaseI-treated total RNA was reverse-transcribed with the High Capacity cDNA Reverse Transcription KIT (Applied Biosystems). The obtained cDNAs were diluted 10-fold, and qRT-PCR assays were performed using a Rotor-Gene 3000 thermal cycler (Corbett Research) and the *Power* SYBR® Green PCR Master Mix (Applied Biosystems). Data were analysed with the Rotor-Gene software (Corbett Research). Expression of the genes used for validation was normalised to the 16 kDa vacuolar ATPase gene. Assays were performed in triplicate for each of the three biological replicates of control and drought-stressed lines. Thus means and standard errors for each selected gene were calculated from nine parallel data points. The primers used for qRT-PCR are listed in Additional file 1.

### Microarray data analysis

For each transcriptome comparison, hybridisations were performed in triplicate for each of the three biological replicates. The data are therefore representative of nine arrays per experiment. Analysis of array images, within- and between-array normalisations were performed as described previously [18]. To identify differentially expressed genes, normalised and log2-transformed data were analysed using the Rank Product method [20], as implemented in the Multiple Experiment Viewer (MeV) software, part of the TM4 Microarray Software Suite [41]. Normalised data were also analysed by two-way ANOVA using the MeV software to determine the genes that were significantly affected by either genotype (wild-type or transgenic) or stress (well-watered or drought) factors. Differentially expressed genes were annotated into functional groups using the MapMan software [42]. Heat maps were created using the MeV software. Microarray data were submitted to ArrayExpress under accession number E-MEXP-3464.

### Metabolite analysis

Sugars and proline were analysed as previously described in a quadrupole-type GC-MS system [18,43], while starch was measured using a previously published method [18].

## Abbreviations

ABA, abscisic acid; ANOVA, analysis of variance; DW, dry weight; GC-MS, gas chromatography-mass spectrometry; qRT-PCR, quantitative real-time polymerase chain reaction; RWC, relative water content; SUS3, sucrose synthase 3; SUS4, sucrose synthase 4; TCA, tricarboxylic acid; TF, transcription factor; TPP, trehalose-6-phosphate phosphatase; TPS1, trehalose-6-phosphate synthase 1; T1, TPS1 transgenic line 1; T2, TPS1 transgenic line 2; T2d, TPS1 transgenic line T2 under drought stress; T2w, TPS1 transgenic line T2 under irrigation; WT, wild-type; WTd, wild-type plant under drought stress; WTw, wild-type plant under irrigation.

## Authors’ contributions

MK performed the microarray experiments and participated in the statistical and bioinformatics analyses; FM participated in the microarray experiments, performed the statistical and bioinformatics analyses, and helped to draft the manuscript. FA validated the microarray results by qRT-PCR. ZJ performed the GC-MS analysis. ZB designed the experiments, coordinated the work and drafted the manuscript. All authors read and approved the final manuscript.

## Supplementary Material

Additional file 1 **PCR primers used for qRT-PCR.** To validate the microarray data, eight genes with differential expression under well-watered and drought conditions in the T2 and WT leaves were selected for qRT-PCR analysis. The following genes were analysed: ribulose bisphosphate carboxylase small chain 2B, fructose bisphosphate aldolase, ETHYLENE-INSENSITIVE3-like 1 transcription factor, a bZIP transcription factor family protein, EMBRYO DEFECTIVE 2220 transcription factor, a plant homeodomain finger family protein, a universal stress protein, and *StDS2*, a drought-inducible potato gene. Gene expression was normalised to the expression of the gene encoding the 16 kDa subunit of the multiheteromeric vacuolar ATPase complex. The file contains the sequences of the primers used in the qRT-PCR analysis. Click here for file

Additional file 2 **Differentially expressed genes in the leaves of wild-type (WT) potato and the*TPS1*transgenic (T2) variant in response to drought stress.** The table contains only those genes that had a statistically significant and greater than 2-fold change in expression, were affected by either genotype (wild-type or transgenic), stress (drought or irrigated) or the interaction of the two factors, and have been annotated into functional groups. For statistical analyses, the “Rank product” and two-way ANOVA methods were used as implemented in the Multiple Experiment Viewer (MeV) software, part of the TM4 Microarray Software Suite [41], while for functional annotation, the MapMan software [42] was employed. Each gene in the table corresponds to an oligonucleotide probe on the POCI potato microarray [19] that can be identified with an identifier number and a gene ID (columns A and B). Columns C to E show the *P*-values of the two-way ANOVA statistics for genes affected by genotype, stress or their interaction. Columns F to I show the log2 value of the expression ratios of particular genes in the different comparisons. T2d, *TPS1* transgenic line T2 under drought stress; T2w, *TPS1* transgenic line T2 under irrigation; WTd, wild-type plant under drought stress; WTw, wild-type plant under irrigation. Columns J to L show functional bin information, gene descriptions and AT numbers. Click here for file

Additional file 3 **Functional classification of the up- and down-regulated genes in T2 and WT potato leaves under drought versus irrigated conditions.** Genes were annotated into functional groups using the MapMan software [42]. Plant labels are as in Additional file 2. Click here for file

Additional file 4 **Heatmap of differentially expressed genes in the “Regulation of transcription” functional group.** The expression ratios of the genes in the T2d versus T2w and WTd versus WTw comparisons are shown as coloured rectangles and were visualised in the Multiple Experiment Viewer (MeV) software. Plant labels are as in Additional file 2. The colour scale indicates the expression ratios as log2 values, with red and green colours for up- and down-regulated genes, respectively. Click here for file

Additional file 5 **Heatmap of differentially expressed genes in the “Signalling” functional group.** Plant and colour labels are as in Additional files 2 and 4, respectively. Click here for file
