# Supplementary material for: Effects of yeast trehalose-6-phosphate synthase 1 on gene expression and carbohydrate contents of potato leaves under drought stress conditions
Source: BMC Plant Biol. 2012 May 30;12:74. doi: 10.1186/1471-2229-12-74 (PMC3459809; doi:10.1186/1471-2229-12-74)
Supplement: Additional file 1 — PCR primers used for qRT-PCR. To validate the microarray data, eight genes with differential expression under well-watered and drought conditions in the T2 and WT leaves were selected for qRT-PCR analysis. The following genes were analysed: ribulose bisphosphate carboxylase small chain 2B, fructose bisphosphate aldolase, ETHYLENE-INSENSITIVE3-like 1 transcription factor, a bZIP transcription factor family protein, EMBRYO DEFECTIVE 2220 transcription factor, a plant homeodomain finger family protein, a universal stress protein, and StDS2, a drought-inducible potato gene. Gene expression was normalised to the expression of the gene encoding the 16 kDa subunit of the multiheteromeric vacuolar ATPase complex. The file contains the sequences of the primers used in the qRT-PCR analysis. [file 1471-2229-12-74-S1.docx]

**PCR primers used in qRT-PCR.**

| **Gene** | **Primer** |
| --- | --- |
| *RbcS-2B* | *F:* ACCTTGACATTACCTCCATTGC  *R:* GCTCGTCAGATAAATCAGGAAGG |
| *FBA* | *F:* CGCAACTAGGGAAATACACCG  *R:* GCTATAAGCTTTTATGCACCGC |
| *EIL1* | *F:* CCGGTTCAATTCTGCTCAAAC  *R:* CATCGAAGAGGAAGCAGTGAC |
| *EMB2220* | *F:* AGTCTGAAGGTGGCAATGAG  *R:* CAGTTGTGGGAGCAAAATG |
| *bZIP* | *F:* TTCTAAAAGTGCCGAGAGTGG  *R:* GCCATTTTGAGATGTGTCAGC |
| *PHD* | *F:* CCTAGTGCAGATAATGGGAGC  *R:* CTTTCATCTGCCAGCTTTGC |
| *USP* | *F:* ATTCTCGTTACTGTTCGCCC  *R:* ACCCCATACCTCTTCATTGTG |
| *StDS2* | *F:* GAGGGAGATGACAACCAGTATG  *R:* CATAGCCACCTTCCTCATTACC |
| *Vacuolar ATPase* | *F:* GTGTCACCTGTTCCATAATTGTG  *R:* CATAATCACGGAGGCCAAAAC |

***Abbreviations:*** *RbcS-2B*, ribulose bisphosphate carboxylase small chain 2B; *FBA*, fructose bisphosphate aldolase; *EIL1*, ETHYLENE-INSENSITIVE3-like 1 transcription factor; *bZIP*, bZIP transcription factor family protein; *EMB2220*, EMBRYO DEFECTIVE 2220 transcription factor; *PHD*, plant homeodomain finger family protein; *USP*, universal stress protein; *StDS2*, drought inducible gene with unknown function; *F*, forward; *R*, reverse
