# Supplementary figures and images for: Effects of yeast trehalose-6-phosphate synthase 1 on gene expression and carbohydrate contents of potato leaves under drought stress conditions
Source: BMC Plant Biol. 2012 May 30;12:74. doi: 10.1186/1471-2229-12-74 (PMC3459809; doi:10.1186/1471-2229-12-74)

## Slide 1
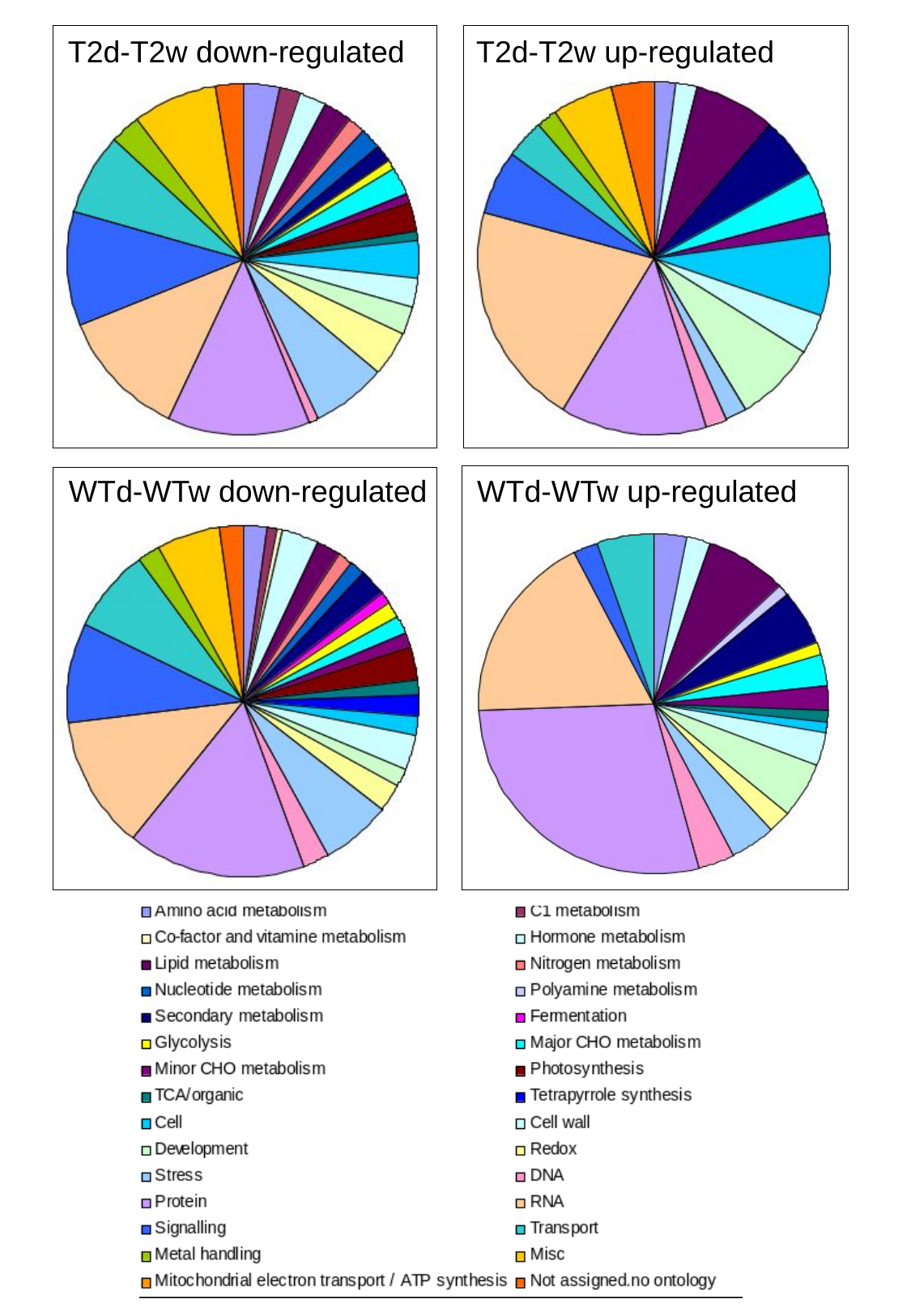

T2d-T2w down-regulated
T2d-T2w up-regulated
WTd-WTw down-regulated
WTd-WTw up-regulated

Supplement: Additional file 3 — Functional classification of the up- and down-regulated genes in T2 and WT potato leaves under drought versus irrigated conditions. Genes were annotated into functional groups using the MapMan software [42]. Plant labels are as in Additional file 2. [file 1471-2229-12-74-S3.ppt]

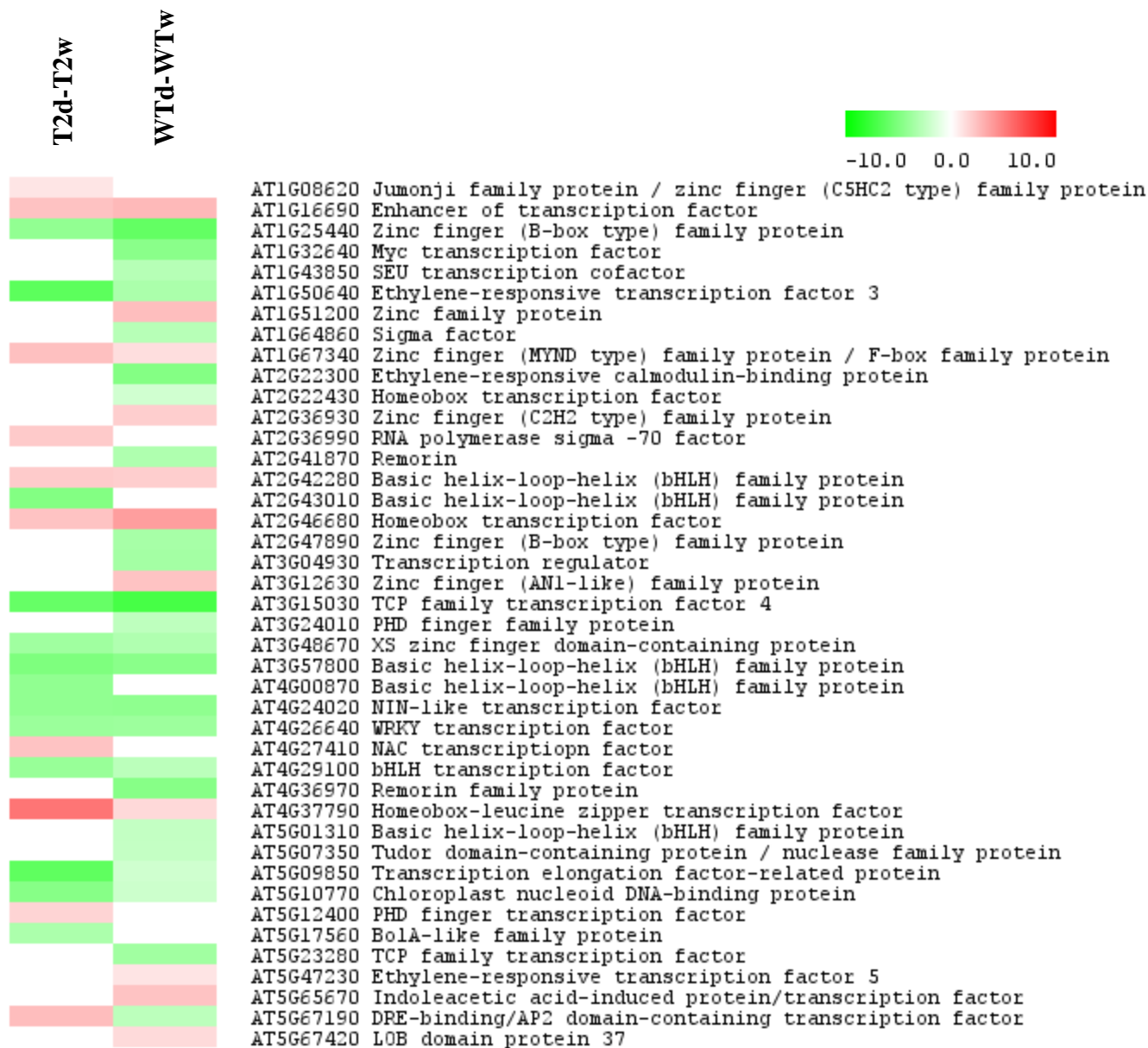

Supplement: Additional file 4 — Heatmap of differentially expressed genes in the “Regulation of transcription” functional group. The expression ratios of the genes in the T2d versus T2w and WTd versus WTw comparisons are shown as coloured rectangles and were visualised in the Multiple Experiment Viewer (MeV) software. Plant labels are as in Additional file 2. The colour scale indicates the expression ratios as log2 values, with red and green colours for up- and down-regulated genes, respectively. [file 1471-2229-12-74-S4.pdf]

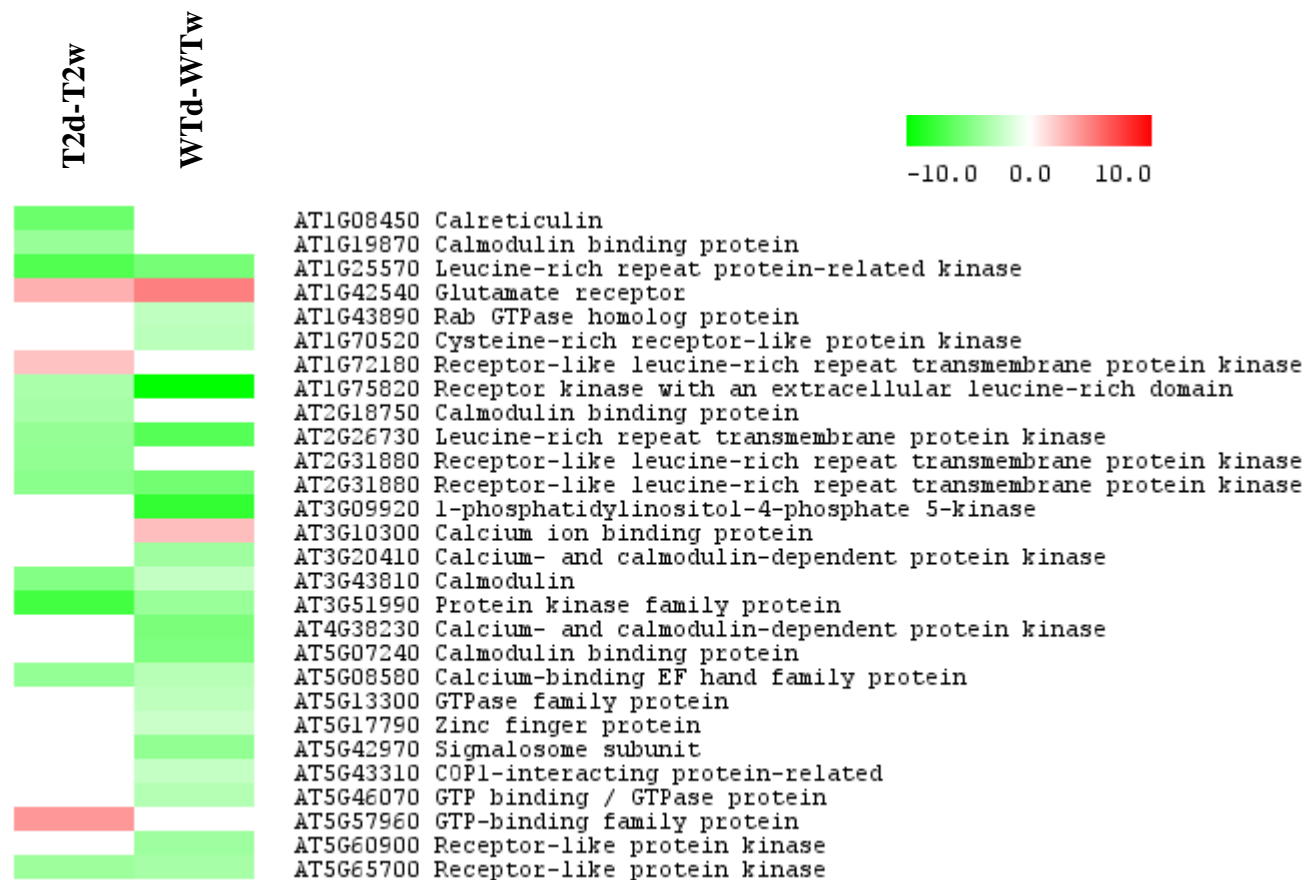

Supplement: Additional file 5 — Heatmap of differentially expressed genes in the “Signalling” functional group. Plant and colour labels are as in Additional files 2 and 4, respectively. [file 1471-2229-12-74-S5.pdf]
